# The G1613A Mutation in the HBV Genome Affects HBeAg Expression and Viral Replication through Altered Core Promoter Activity

**DOI:** 10.1371/journal.pone.0021856

**Published:** 2011-07-21

**Authors:** Man-Shan Li, Terrence Chi-Kong Lau, Sophie Ka-Ping Chan, Chi-Hang Wong, Patrick Kwok-Shing Ng, Joseph Jao-Yiu Sung, Henry Lik-Yuen Chan, Stephen Kwok-Wing Tsui

**Affiliations:** 1 School of Biomedical Sciences, The Chinese University of Hong Kong, Shatin, N.T., Hong Kong SAR, China; 2 Department of Medicine and Therapeutics, The Chinese University of Hong Kong, Shatin, N.T., Hong Kong SAR, China; 3 Centre for Microbial Genomics and Proteomics, The Chinese University of Hong Kong, Shatin, N.T., Hong Kong SAR, China; 4 Department of Biology and Chemistry, City University of Hong Kong, Kowloon, Hong Kong SAR, China; Yonsei University, Korea, Republic of

## Abstract

Infection of hepatitis B virus (HBV) causes acute and chronic hepatitis and is closely associated with the development of cirrhosis and hepatocellular carcinoma (HCC). Previously, we demonstrated that the G1613A mutation in the HBV negative regulatory element (NRE) is a hotspot mutation in HCC patients. In this study, we further investigated the functional consequences of this mutation in the context of the full length HBV genome and its replication. We showed that the G1613A mutation significantly suppresses the secretion of e antigen (HBeAg) and enhances the synthesis of viral DNA, which is in consistence to our clinical result that the G1613A mutation associates with high viral load in chronic HBV carriers. To further investigate the molecular mechanism of the mutation, we performed the electrophoretic mobility shift assay with the recombinant RFX1 protein, a trans-activator that was shown to interact with the NRE of HBV. Intriguingly, RFX1 binds to the G1613A mutant with higher affinity than the wild-type sequence, indicating that the mutation possesses the trans-activating effect to the core promoter via NRE. The trans-activating effect was further validated by the enhancement of the core promoter activity after overexpression of RFX1 in liver cell line. In summary, our results suggest the functional consequences of the hotspot G1613A mutation found in HBV. We also provide a possible molecular mechanism of this hotspot mutation to the increased viral load of HBV carriers, which increases the risk to HCC.

## Introduction

Hepatitis B virus (HBV) infection is a considerable burden to health in the Asian-Pacific region. It causes acute and chronic hepatitis which is closely associated with the development of cirrhosis and hepatocellular carcinoma (HCC). Approximately 60–80% of world's HCC is related to HBV. It is estimated that chronic HBV carriers would have 100 times higher risk developing HBV-related HCC compared to uninfected individuals [1]. HBV is classified into 8 genotypes (A–H) with distinct geographical distribution and can be further divided into a total of 24 subgenotypes [2], [3]. Genotypes B and C are predominant in South-east Asia. In East (Korea and Japan) and northern China, HBV subgenotype Ce is more prevalent whereas subgenotype Cs is usually found in Southeast Asia, including Vietnam, Thailand, Malaysia, and southern China [4].

**Figure 1:**
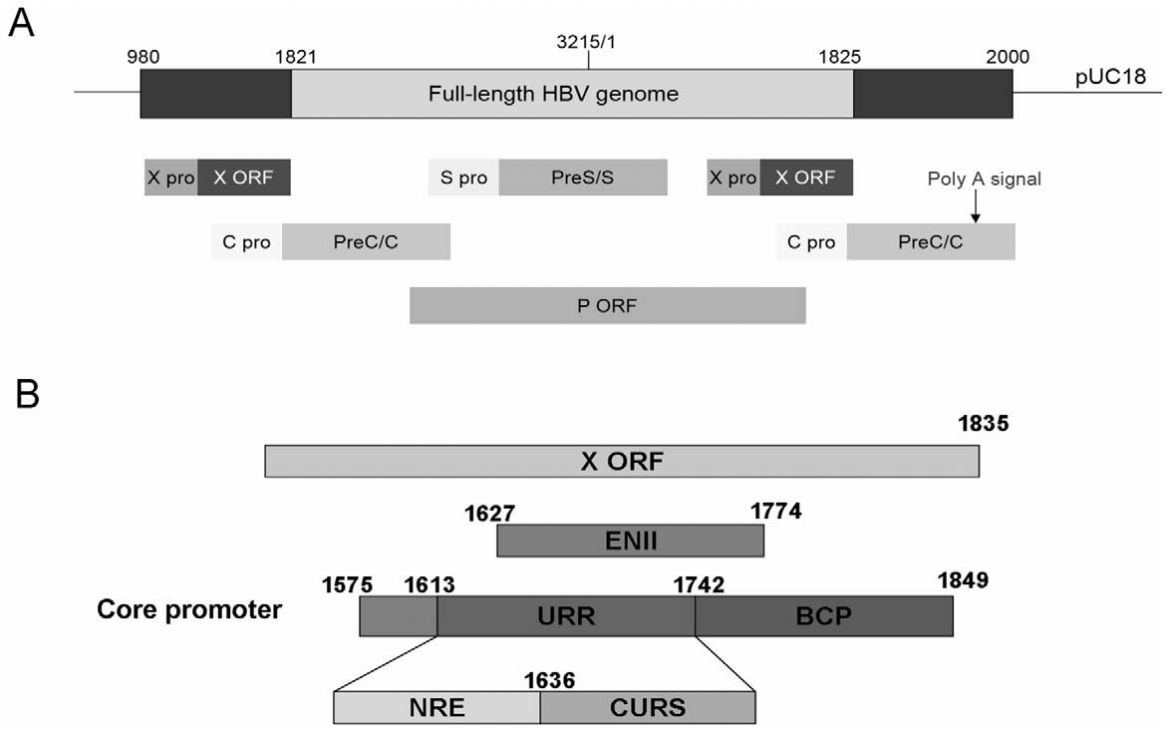
Schematic diagrams of HBV genome and core promoter. (**A**) The genome organization of 1.3× HBV genome used in this study (nt. 980–2000). The numbering of the nucleotide start at the unique *EcoR*I site as ‘1’ indicated in the middle of the genome. The two ends contains repeated parts from nt. 980–1821 and nt. 1825–2000 (dark grey colour) including the polyadenylation signal located downstream of the core promoter. The whole genome was cloned into pUC18 vector. The genes in the genome are partially overlapping. ORF: open reading frame; pro: promoter; X: region coding for X protein; PreC/C: precore/core region coding the precore and core protein; PreS/S: region coding for large, middle and small surface proteins; P: region coding for viral polymerase. (**B**) The major elements in HBV core promoter. The core promoter overlaps with 5′ end of X ORF and enhancer II (ENII). The activity of the basal core promoter (BCP) is regulated by the upper regulatory region (URR), which is further divided into negative regulatory element (NRE) and the core upstream regulatory sequence (CURS).

HBV DNA is a relaxed circular, partially double-stranded molecule of 3.2 kb. It contains four partially overlapping open-reading frames (ORFs) which codes for seven proteins. The PreC/C ORF encodes for the precore and core proteins. The precore protein is posttranslationally modified to form the secretory e antigen (HBeAg) whereas the core protein is the structural protein which composes the capsid of the virus. The polymerase (P) ORF encodes for the viral polymerase-reverse transcriptase. The preS/S ORF encodes for various surface proteins whereas the X ORF encodes for a transcriptional transactivator X protein (HBx). One of the characteristics of HBV genome is the partially overlapping of the genes. In order to reduce the disruption of the genes in the HBV genome, 1.3× HBV genomes was often used for *in-vitro* studies [5]. The organization of the genome is depicted in Figure 1A.

HBV is hepatotrophic due to its specific interaction of various liver-enriched transcription factors such as HNF-4, HNF-3 and C/EBP [6]. The HBV core promoter is essential to the viral replication, as it regulates the transcription of the pregenomic RNA (pgRNA) and precore (preC) mRNA. The pgRNA is then translated to core protein and the viral polymerase, as well as being a template for viral replication, whereas the preC mRNA codes for a precursor protein of HBeAg which is a serological marker used for HBV infection diagnosis [7].

The core promoter is composed of two regions, the basal core promoter (BCP) and the upper regulatory region (URR) [8] (Figure 1B). The BCP (nt. 1742–1849) region contains *cis*-acting element which initiates the transcription of pgRNA and preC mRNA [9]. Although both transcripts are driven by the same promoter, their transcriptions are differentially regulated [10]. In BCP region, a double mutation, A1762T/G1764A, is commonly found to associate with HBV genotype C [11], which affects the viral replications. The URR consists of the negative regulatory element (NRE, nt. 1613–1636) and the core upstream regulatory sequence (CURS, nt. 1637–1742) (Figure 1B). Both elements overlap the enhancer II (ENII, nt. 1627–1774) that recruits liver-enriched transcription factors for its activity. The CURS can activate the BCP in well-differentiated hepatoma cells through *cis*-acting elements immediately upstream of BCP in an orientation- and position-dependent manner [12], [13]. Nevertheless, the stimulatory effect of ENII and CURS can be repressed by the effect of upstream NRE [12]. The NRE is composed of three subregions: NREα, NREβ and NREγ [14]. The RFX1, a transactivating protein, was demonstrated to bind the NREγ region but not in randomly introduced NREγ mutants [14]. Moreover, the sequence of NREγ shares 85% similarity with the RFX1 consensus binding sequences, suggesting that RFX1 is one of the transactivators that regulate the viral replication of HBV [15], [16], [17].

Recently, several factors and *cis*-acting elements were shown to involve in the posttranscriptional control of the HBV gene expression. Posttranscriptional regulatory elements (PRE) have been found at the 3′ end of all HBV transcripts as they terminate at a common polyadenylation site [18]. The PRE was predicted to contain multiple functional conserved elements at nucleotide positions 1151–1410, 1411–1433 and 1510–1620. Although the exact role of the PRE has yet to be elucidated [15], [16], PRE have been shown to be required for efficient HBV surface protein expression by facilitating the cytoplasmic localization of surface protein mRNA [19], [20], and contribute to the HBV RNA stability through the interaction with human La protein [21], [22], [23], [24], [25]. In addition, although splicing of HBV RNA is not required to produce viral proteins, spliced variants of HBV RNAs have been detected in sera and in liver samples *in vivo* and *in vitro* [20]. A splicing regulatory element on PRE is found in stimulating the splicing as a result of RNA-protein and protein-protein interaction [26]. However, it remains unclear if splicing variants have any important role in the life cycle of HBV.

In our previous study [27], the G1613A mutation was identified as a hotspot in HCC patients. Coincidently, the mutation is situated on the NREγ on core promoter, as well as the conserved region within PRE on HBV RNAs. In this study, we set out to elucidate the functional consequences of the G1613A mutation on the synthesis of HBV serological markers, HBeAg and HBV surface antigen (HBsAg), as well as the production of viral DNA and RNA. We showed that the G1613A mutation is associated to high viral load in HBV carriers with statistically significance. Moreover, this mutation enhanced the HBV viral DNA production and suppressed the secretion of HBeAg. Intriguingly, the hotspot mutation locates in the RFX1 consensus binding sequence of the NREγ region. We further showed that RFX1 transactivates the core promoter activity by interacting to the NREγ region with specificity to the mutation. Overall, our data demonstrated the functional consequences of the G1613A mutation, and suggested a possible molecular link between the mutation and the resulted phenotype of the virus.

## Materials and Methods

### Patients in the study of viral load and G1613A mutation

Residual serum samples from a cohort of chronic hepatitis B patients undergoing a surveillance program for hepatocellular carcinoma recruited from the Hepatology Clinic, Prince of Wales Hospital from October 1997 to November 2000 was studied [28]. Based on sequencing of the HBV S gene, 255 chronic carriers were confirmed to have subgenotype Cs HBV infection. The configuration of nt. 1613 was determined in the available residual serum samples of these patients by direct DNA sequencing.

### Alignment of HBV NRE sequences of genotype C

The HBV sequences were obtained from National Center for Biotechnology Information (GenBank) database (http://www.ncbi.nlm.nih.gov/). A total of 805 HBV sequences of genotype C were aligned within the nt. 1604–1636. The counts of each base within nt. 1611–1619 were calculated.

### Construction of promoter clones from patient isolates

Constructs of HBV core promoters (nt. 1575–1813) were derived from HBV isolates extracted from patients' serum. The original configuration of nt. 1613 on the core promoter isolated from the carrier was ‘G’, and it was mutated to ‘A’ by site-directed mutagenesis. These constructs are belonged to HBV subgenotype Cs. The HBV preS1 promoter, of nucleotide 2706–2887, was used as control. The purified PCR products were cleaved by *Xho*I and *Hind*III (Amersham Biosciences). After purification, the digested product were ligated into pGL3-basic vector (Promega) and finally cloned and purified by Midi Kit (Qiagen) according to manufacturer's instruction. The sequences of all the constructs were confirmed by DNA sequencing.

### Construction of 1.3× HBV genomes of G1613A mutants with/without the BCP mutation

HBV DNA of subgenotype Cs extracted from a chronic carrier was used as template. The 1.3× HBV constructs was designed to follow the organization of the 1.5× HBV clone (genotype A) provided from VIDRL, only with a shorter genome length. It starts from the X promoter (nt. 980) and extended more than one genome length to include two copies of X ORF. It ends 80 base pairs downstream of the poly(A) signal to include the *cis*-regulatory elements for effective replication (nt. 2000). The G1613A mutation and the BCP mutation were introduced into both 5′ and 3′ copy of the core promoter in the genome by site directed mutagenesis.

### Transfection of 1.3× HBV genomes

Human hepatoma cells HuH7 purchased from Japanese Collection of Research Bioresources Cellbank (JCRB) (cell number: JCRB0403) were seeded on 60-mm dishes in 5 ml of complete DMEM to obtain approximately 60% confluent on the day of transfection. The 1.3× HBV genome, together with pSEAP-Control plasmid (secreted human placental alkaline phosphatase as transfection efficiency control) was transfected using FuGENE 6 (Roche Applied Science). Growth media were changed 2 days after transfection. The transfection efficiency was normalized by the secreted alkaline phosphatase (SEAP) activity in the culture media. The SEAP Reporter Gene Assay (Roche Applied Science) was performed according to the manufacturer's instruction.

### Measurement of HBeAg and HBsAg levels

Fifty microliters of supernatant and cell lysate was collected for extracellular and intracellular HBeAg and HBsAg detection respectively by Microparticle Enzyme Immunoassay (AUTOBIO Diagnostics Co. Ltd) according to manufacturer's instruction. All readings exceeded the cut off value were interpreted as positive. They were normalized to the result of SEAP reporter assay as transfection efficiency control.

### HBV DNA extraction

Intracellular HBV DNA was prepared by adding 800 µl lysis buffer (10 mM Tris pH 7.5, 1 mM EDTA, 50 mM NaCl and 0.5% Nonidet P-40) to the cells at 4°C for 15 min. Cells lysates were spun at 8,000× g for 5 min to remove the nuclei. Transfected plasmid DNA was eliminated from cell lysate by treatment with DNaseI (20 U) and MgCl_2_, and incubated at 37°C for 2 hr. Then followed by adding 5× stop buffer [2.5% (w/v) sodium dodecyl sulphate (SDS), 100 mM Tris pH 7.5 and 125 mM EDTA] for inactivation and proteinase K (Invitrogen) to a final concentration of 0.5 mg/ml. The samples were incubated at 37°C overnight for complete digestion of the core particles and release of HBV DNA. Extracellular HBV DNA was collected in supernatant at 5-day posttransfection. The core particles in the supernatant were precipitated by 26% polyethylene glycol buffer (Sigma) at 4°C overnight, and spun at 16, 000× g for 20 min at 4°C. The pellet was then resuspend in 200 µl virus buffer (10 mM Tris pH 7.5 and 5.5 mM MgCl_2_), then lysed by 5× stop buffer and treated with proteinase K as the intracellular HBV DNA preparation. Afterwards, the intracellular and extracellular HBV DNA was extracted using Qiamp DNA mini kit (Qiagen) according to manufacturer's instructions. DNA was finally eluted into 50 µl of nuclease-free water.

### Real-time quantitative PCR of HBV DNA

Two microliters of 50-fold diluted DNA was used in 10 µl real-time PCR reaction mixture with 1× SYBR-green power master mix and 7500 Fast Real-Time PCR System (Applied Biosystems). Intracellular and extracellular HBV DNA was detected by primers according to Mendy *et al.* [16]. Each test run included positive and negative controls. The reaction condition was modified to 95°C for 10 min followed by 40 cycles of 95°C for 15 s and 61.5°C for 1 min. The level of HBV DNA was expressed in relative to the corresponding wild-type G1613 construct.

### Southern blot and HBV DNA hybridization

The amount of HBV DNA extracted from both intracellular and extracellular cores from each plate of transfected cells was normalized using the SEAP activity as an internal control as reported previously [29]. After electrophoresis in a 1.5% agarose gel, HBV core DNA was blotted onto a nylon membrane. Twenty-five nanograms of full-length HBV DNA (subgenotype Cs) was labeled by random primer with [^32^P]dCTP and used as the probe for hybridization. The blots were hybridized at 42°C overnight and followed by washing. The membranes were exposed to phosphorimager plates and scanned by phosphorimager reader. Densitometry of each lane was performed for semi-quantification of the intensity of signals.

### Northern blot analysis

Total cellular RNA was extracted from transfected cells with Trizol reagent as described above. Following the standard method [26], 10 µg of total RNA was separated on agarose/formaldehyde gels, transferred onto nylon membrane and fixed by UV irradiation. Hybridization and exposure were similar to that described for Southern blotting. To normalize to the transfection efficiency, the amount of RNA was calibrated according to the relative SEAP activities.

### Construction of pCMV-TNT-RFX1 clone

The cDNA clone of *Homo sapiens* regulatory factor X1 (RFX1) was purchased from OriGene Technologies, Inc. (catalogue no. RC207872). The plasmid has been derived from single clone *E. coli* cultures and purified as 10 µg transfection-ready dried plasmid DNA. The plasmid contained the full-length RFX1 ORF with a myc-tag at the C-terminal cloned into the pCMV6 Entry vector (pCMV-RFX1). The sequence of this clone matched the reference sequence published in the National Center for Biotechnology Information with accession no. NM_002918.3. The full-length ORF of RFX1 in pCMV-RFX1 plasmid was subcloned into pCMVTNT™ Vector (Promega), which is designed for the convenient expression of cloned genes using *in vitro* expression systems. The PCR reaction included 0.2 µM each of forward and reverse primers, 1 µl of 1000-fold diluted pCMV-RFX1 and 25 µl of 2× PicoMaxx mastermix in a total volume of 50 µl. The PCR was performed with a 3 min initial denaturation at 94°C, followed by 32 cycles of amplification (94°C for 36 s, 58°C for 30 s and 72°C for 3 min) and a final extension at 72°C for 10 min. The PCR products were examined in 1% TAE agarose gel and purified by gel extraction kit (Qiagen), followed by *Xho*I and *Mlu*I digestion in 1× NEB buffer 3 at 37°C for 16 hr. After that, the digested fragment was ligated to *Xho*I/*Mlu*I digested pCMV-TNT empty vector and subsequently transformed to *E. coli* and purified. The DNA sequences of the RFX1 construct (pCMV-TNT-RFX1) was confirmed by DNA sequencing.

### *In vitro* transcription/translation of RFX1 protein

The RFX1 gene was *in vitro* transcribed and translated using TNT® Quick Coupled Transcription/Translation Systems (Promega) according to manufacturer's instruction. In brief, the reaction contained 40 µl of Quick TNT master mix, 2 µl of radioactive ^35^S-methionine (1000 Ci/mmol) (Perkin Elmer) or 20 µM of methionine, and 1–5 µg of DNA in 50 µl of reaction. The mixture was incubated at 30°C for 90 min and the labeled translation products were analyzed by 10% SDS-PAGE. Then, the gel was blotted on 3 mm Whatman paper and dried at 75°C for 1 hr, followed by signal detection by autoradiography or phosphorimager.

### Electrophoretic mobility shift assay (EMSA)

To find out endogenous nuclear proteins in HuH7 that bound to the NRE region of the core promoter. G1613 (wild-type) (sense 5′-GCACGTCGCATGGAGACCACCGTGAACGCGC-3′; antisense 5′-GGCGTTCACGGTGGTCTCCATGCGACGTGC-3′) and A1613 (mutant) (sense 5′- GCACGTCGCATGGAAACCACCGTGAACGCC-3′; antisense 5′- GGCGTTCACGGTGGTTTCCATGCGACGTGC-3′) annealed NRE probes were used. The sequences of the probes are the same except for the nt. 1613 (underlined). Nuclear and cytoplasmic extracts of HuH7 cells were isolated by NE-PER Nuclear and Cytoplasmic Extraction Reagents (PIERCE Biotechnology) according to manufacturer's instruction. The protein was aliquoted and kept at −80°C until use. Annealed G1613 (wild-type) and A1613 (mutant) NRE probes and annealed non-specific oligos (sense 5′- GGAATTACGTGGCCACTCGAGGGAATTACG-3′; antisense 5′- CGTAATTCCCTCGAGTGGCCACGTAATTCC-3′) were end-radiolabeled with ^32^P by T4 kinase (GE Healthcare). Unincorporated nucleotide was removed by G-25 column (GE Healthcare) according to manufacturer's instruction. The DNA binding reactions contained 5–20 µg of protein, 1 µl of labeled probe, 2 µl of 10× binding buffer (500 mM NaCl, 100 mM TrisHCl pH 7.5, 25 mM MgCl_2_, 40% Glycerol, 5 mM DTT), 1 µl of 1% NP40 and 1 µl of poly (dI · dC) (GE Medical Systems) in a reaction volume of 20 µl. The reaction was incubated at room temperature for 1 hr and then analyzed in 5% native polyacrylamide gel. After that, the gel was blotted to a filter paper and dried at 75°C for 1 hr, followed by signal detection by autoradiography or phosphorimager. To validate the specificity of the binding, up to 250× of cold probes were added to the binding reaction before the addition of the labeled probes. The difference in the binding affinities was reflected by the relative intensity of the bands.

### Co-transfection of pCMV-RFX1 with HBV promoters

HuH7 cells were cultured in Dulbecco's Modified Eagle Medium (DMEM) (Sigma-Aldrich) supplemented with 10% fetal bovine serum (FBS) (Hyclone) and 1% streptomycin-penicillin (Invitrogen) at 37°C and 5% CO_2_. Cells were seeded on 60-mm plate to obtain approximately 60% confluent on the day of transfection. Then, 0.6 µl of FuGENE6 was diluted in a dropwise manner in 20 µl of plain medium and incubated at room temperature for 5 min. Afterwards, 0.1 µg of each of pCMV-RFX1 plasmid and pGL3-HBV promoter plasmid and 0.001 µg of pCMV-PRL plasmid were added to the diluted FuGENE6 and incubated at room temperature for 20 min. Then, 20 µl of mixture was added into cells and incubated at 37°C in 5% CO_2_ incubator for 2 days. Dual luciferase reporter assay was performed according to manufacturer's instruction.

### Dual-luciferase reporter assay

Cells were harvested at 48 hr after transfection. After washing with phosphate buffer saline (PBS), cells were lysed with 400 µl of 1× Passive Lysis Buffer (Promega) for 15 min at room temperature. Cell lysates were centrifuged at 10,000× g for 30 s to remove cell debris and nuclei. Luciferase assay was performed according to manufacturer's instruction (Promega). The results were shown as relative ratio of the Firefly luciferase activities normalized to the *Renilla* luciferase activities in triplicates (mean value ± S.D).

### Data analysis

Data was analyzed by the software SPSS version 11.5 for Windows. Continuous variables were expressed as mean with standard deviation, and compared by *t* test or Mann-Whitney *U* test as appropriate. Categorical variables were compared by Chi-square test or Fisher's exact test as appropriate. All statistical tests were 2-sided, and *P* values less than .05 will be considered as statistically significant.

## Results

### The higher prevalence of the G1613A mutation in HCC patients

In our previous study, we showed the G1613A mutation is a hotspot mutation in HBV subgenotype Cs in HCC patients [26]. Here, we further evaluate the prevalence of the mutation in HBV genotype B and subgenotype Ce. The HBV sequences were obtained as previously described and sequenced [30]. We showed that the G1613A mutation was found with higher prevalence in HCC patients in all HBV genoytpes (Table 1). Intriguingly, the mutation is associated to HCC in subgenotype Cs (36.2%, *P*<.003) with statistically significance, suggesting that the mutation may play a role in a genotype-dependent HBV-related HCC pathogenesis. In order to further characterize the mutation in genotype C HBV genome, we aligned 803 HBV complete genome sequences obtained from National Center for Biotechnology Information (GenBank). We found the nt. 1613 is the only hotspot in the NRE region from nt. 1604–1636. The summarized result of alignment from nt. 1611–1619 was shown in Table 2, in which 26% of the NRE sequences contain G1613A mutation.

**Table 1:** The prevalence of G1613A mutation in HBV carriers and HCC patients.

HBV Genotype/subgenotype	Cases	Number of cases	Nucleotide at 1613			## *P* value
			G(%)	A(%)	#R (%)	
B (N = 87)	Carriers	50	39 (78)	11(22.0)	0 (0)	.175
	HCC	37	24 (64.9)	13 (35.1)	0 (0)	
Cs (N = 86)	Carriers	39	33 (84.6)	4 (10.3)	2 (5.1)	* .003
	HCC	47	29 (61.7)	17 (36.2)	1 (2.1)	
Ce (N = 26)	Carriers	10	9 (90.0)	1 (10.0)	0 (0)	.179
	HCC	16	9 (56.3)	6 (37.5)	1 (6.3)	

*The G1613A mutation was found significantly higher prevalent in HCC patients in subgenotype Cs.

# R represents purines, A or G.

## P value was determined using chi's square analysis in SPSS software. A *P*-value of .05 or less is regarded as statistically significant.

**Table 2:** Analysis of 803 NRE sequences of HBV genotype C.

Position	Nucleotide on HBV NRE region	Count	%
1611	G	803	100.0
1612	A	795	99.0
	C	8	1.0
a1613	G	583	72.6
	A	209	26.0
	R	11	1.4
1614	A	798	99.4
	G	5	0.6
1615	C	803	100.0
1616	C	803	100.0
1617	A	799	99.5
	T	2	0.2
	C	2	0.2
1618	C	803	100.0
1619	C	803	100.0

a The nucleotide 1613 is the only hot spot mutation within nt. 1611–1619. The HBV NRE sequences were obtained from National Center for Biotechnology Information (GenBank) database (http://www.ncbi.nlm.nih.gov/).

### Association of the prevalence of G1613A mutation to high viral load in chronic HBV carriers

Previously, a clinical scoring system for HCC risk assessment suggested that the serum viral load more than 6 log copies/ml is associated with higher risk in developing HCC in both univariate and multivariate analysis [27]. To characterize the relationship between the HBV viral load and the G1613A mutation, we quantified the serum viral load in 255 chronic HBV carriers as described in the previous study [12]. We also sequenced the NRE region of HBV genomes obtained from their serum. As shown in Table 3, 129 (41 females and 88 males) chronic carriers had their serum viral load more than 6 log copies/ml. Moreover, the prevalence of the G1613A mutation is significantly associated to higher viral load in female carriers in a univariate analysis. Although the discrepancy between sexes is unclear, the analysis suggests that high viral load could be one of the consequences of the G1613A mutation in HBV.

**Table 3:** The relationship between the prevalence of G1613A mutation and viral load in HBV chronic carriers of subgenotype Cs.

Cases	Viral load	Nucleotide at 1613				# *P* value	##Harzard Ratio
(N = 255)	(log copies/ml)	G	(%)	A	(%)		(95% CI)
Male (N = 167)	<6	62	(78.5)	17	(21.5)	.589	0.81 (0.378–1.737)
	≥6	72	(81.8)	16	(18.2)		
Female (N = 88)	<6	40	(85.1)	7	(14.9)	*.01	3.657 (1.32–10.133)
	≥6	25	(61.0)	16	(39.0)		

*The G1613A mutation is associated with higher serum viral load in female carriers.

# P value was determined using chi's square analysis in SPSS software. A *P*-value of .05 or less is regarded as statistically significant.

## Harzard Ratio is an estimation of relative risk.

### G1613A mutation drastically suppresses HBeAg secretion and enhances viral DNA production *in vitro*

**Figure 2:**
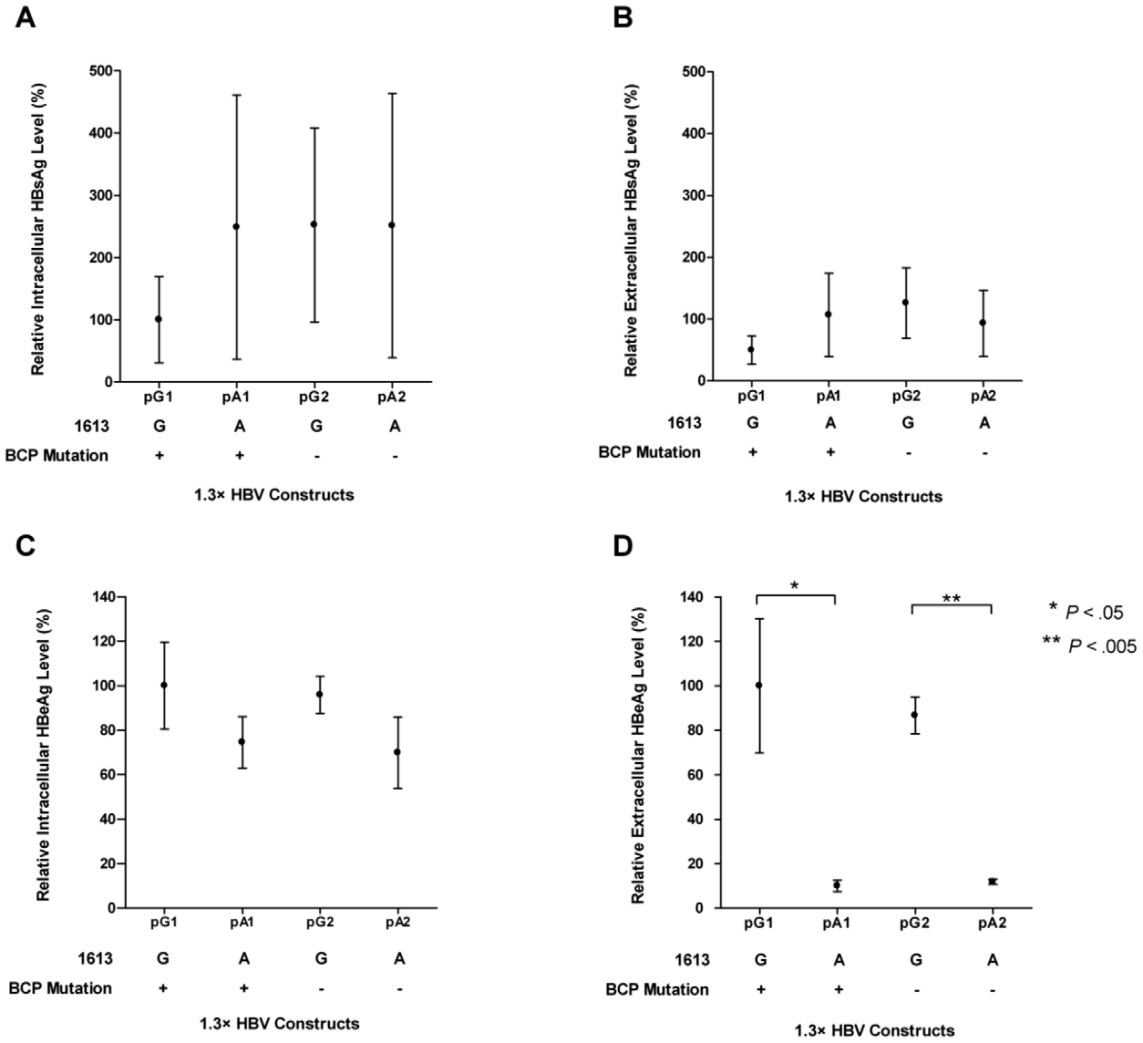
The effect of G1613A mutations on the levels of HBsAg and HBeAg. (**A**) Intracellular level of HBsAg, (**B**) extracellular level of HBsAg, (**C**) intracellular level of HBeAg and (**D**) extracellular level of HBeAg were measured after transfection with 1.3× HBV genomes with indicated mutations into HuH7 cells by ELISA as described in materials and methods. All the constructs have the same genetic backgrounds except the indicated mutations. The mutations were introduced into the HBV genome by site directed mutagenesis. The levels of the antigens were normalized to the transfection efficiency measured as the co-transfected SEAP activities. The decrease with statistical significance in the HBeAg in the extracellular media is indicated by asterisk(s). Data were presented as normalized values in 4 independent experiments (mean ± S. D.).

**Figure 3:**
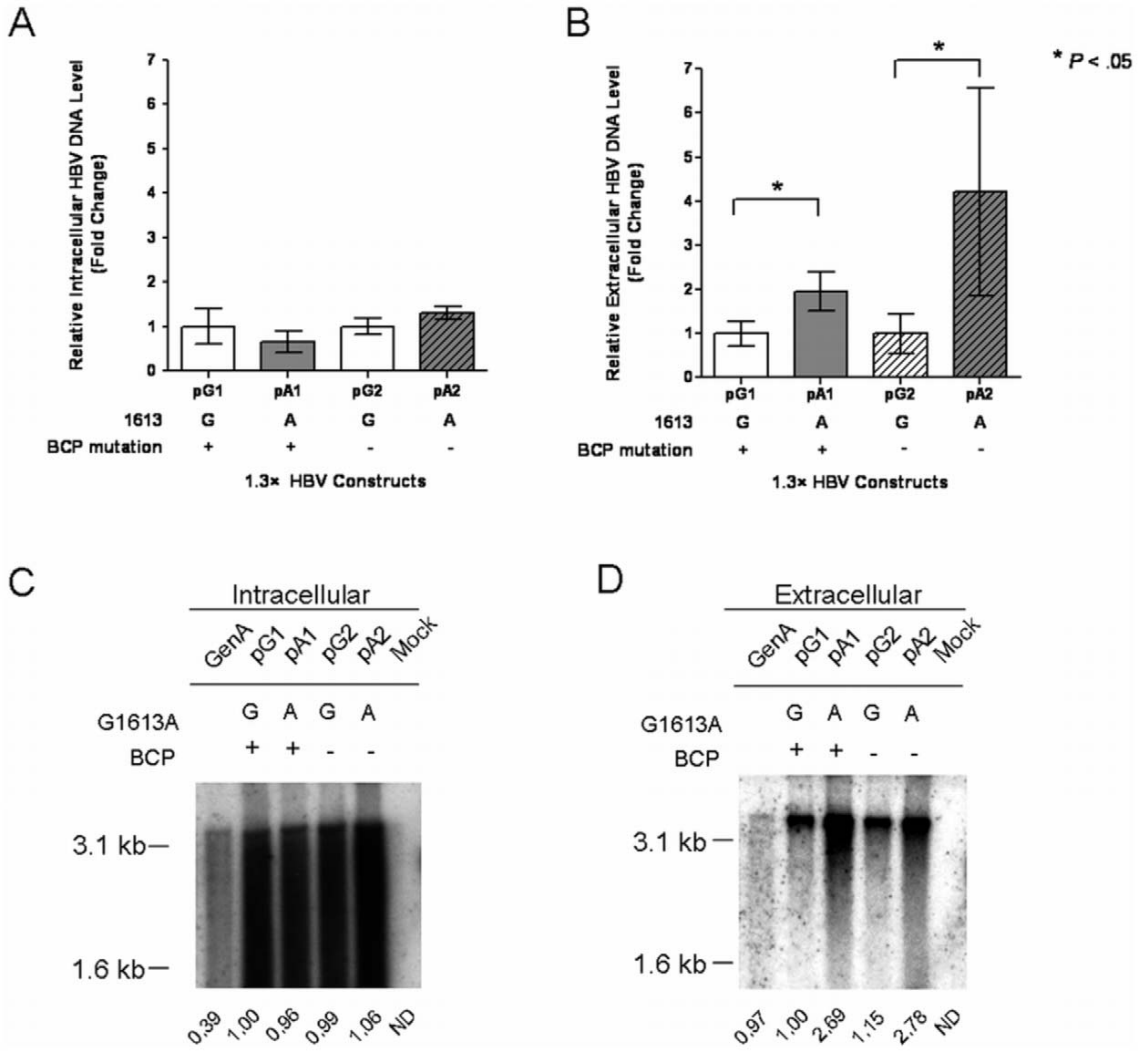
The effect of G1613A mutation on the HBV DNA level. The relative intracellular and extracellular HBV DNA levels were measured by quantitative real-time PCR (**A** and **B**) and Southern blot analysis (**C** and **D**) respectively after transfection with 1.3× HBV genomes into HuH7 cells. Except the indicated mutations, all the constructs have the same genetic background. Results in real-time PCR were normalized to the wild-type and shown in mean values ± S.D. from 3 independent experiments. The increases of extracellular HBV DNA level with statistical significance are indicated by asterisks. In Southern blot analysis, the amount of HBV DNA extracted from both intracellular and extracellular cores particles from each plate of transfected cells was normalized using the SEAP activity as an internal control. The relative signals were quantified by densitometer and shown below each graphs for comparison. GenA: Huh7 cells transfected with a replicative competent 1.5× HBV plasmid (Genotype A) as positive control. ND: not determined.

To demonstrate the effect of the mutations on the HBsAg and HBeAg production, 1.3× HBV genomes of subgenotype Cs with the same genetic background but different combination of the G1613A and BCP mutations were constructed (pG1, pA1, pG2 and pA2) and transfected into HuH7 cells. Since the BCP mutation has high prevalence in HCC patients, it is used (pG1 and pA1) as an independent control to evaluate the effect of G1613A mutation. The viral HBsAg and HBeAg level in the intracellular and extracellular compartments were measured. As shown in Figure 2, the HBsAg levels in both intracellular and extracellular compartments, as well as the intracellular level of HBeAg were not affected by any mutations introduced (Figure 2A, 2B and 2C). In contrast, the G1613A mutation significantly decreased the extracellular HBeAg level by 90% (pA1 *vs* pG1) and 86% (pA2 *vs* pG2) (Figure 2D). Next, the core-particle associated HBV DNA in both intracellular and extracellular compartments were measured by quantitative real time PCR. The DNA levels in A1613 mutants were normalized to that in G1613 wild-type. As shown in Figure 3A, insignificant change is observed in the level of intracellular HBV DNA in response to the mutations. On the other hand, G1613A mutation significantly increased the level of extracellular HBV DNA by 2 folds (pA1 *vs* pG1) and 4 folds (pA2 *vs* pG2) respectively (Figure 3B). To further confirm the enhanced viral DNA production in the mutants, Southern blots were performed. As shown in Figure 3C and 3D, the level of intracellular HBV DNA intermediates was not affected whereas the level of extracellular HBV DNA significantly increased in G1613A mutants. These results suggest that the G1613A mutation suppressed the HBeAg secretion and enhance total HBV DNA synthesis.

### Effect of G1613A mutation on HBV transcripts

**Figure 4:**
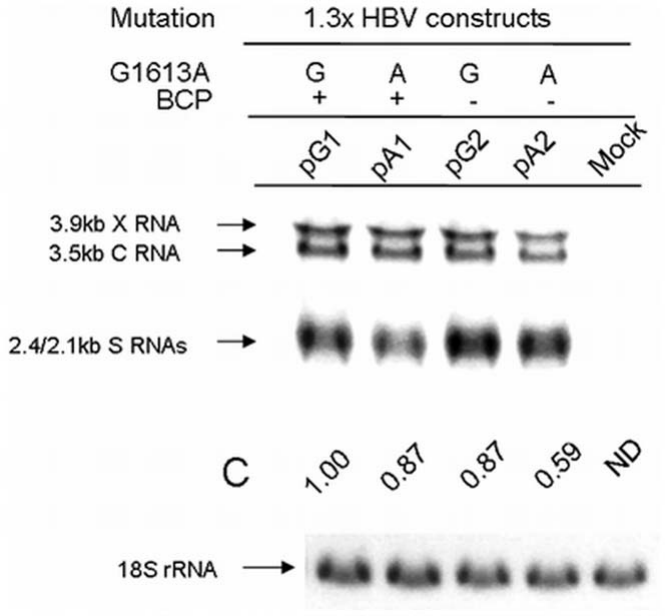
Northern blot analysis of HBV RNA. HuH7 were transfected with 1.3× HBV genomes with indicated mutations. All the constructs have the same genetic background except the indicated mutations. Total RNA was extracted at 5-day posttransfection.10 µg of total RNA were loaded in each lane. X, C and S indicate the 3.9-kb X gene transcript, the C gene transcripts and the S gene transcripts, respectively. The relative signals of the total core RNA (C) were quantified by densitometer and showed below for comparison. The 18S rRNA showed at the bottom is the internal control of RNA loading. ND: not determined.

To further investigate the functional effect of the G1613A mutation on viral transcription, Northern blot analysis was performed to measure the relative amount of the HBV transcripts after the transfection of the 1.3× HBV genomes (Figure 4). The level of HBV RNA transcription does not change significantly when compared with HBeAg and HBV DNA levels, suggesting that the G1613A mutation may exert its effect at the posttranscriptional regulation level. In fact, the G1613A mutation locates within the conserved regions of PRE on HBV RNAs. One of the possible reasons for the subtle change of the total core RNA level is likely due to the combined effect of enhancement of promoter activity and the involvement of the posttranscriptional element (PRE) effect at the 3′end of HBV transcript that affects the RNA stability. We described in detail in the discussion section.

### G1613A mutation affects nuclear proteins binding on NRE

**Figure 5:**
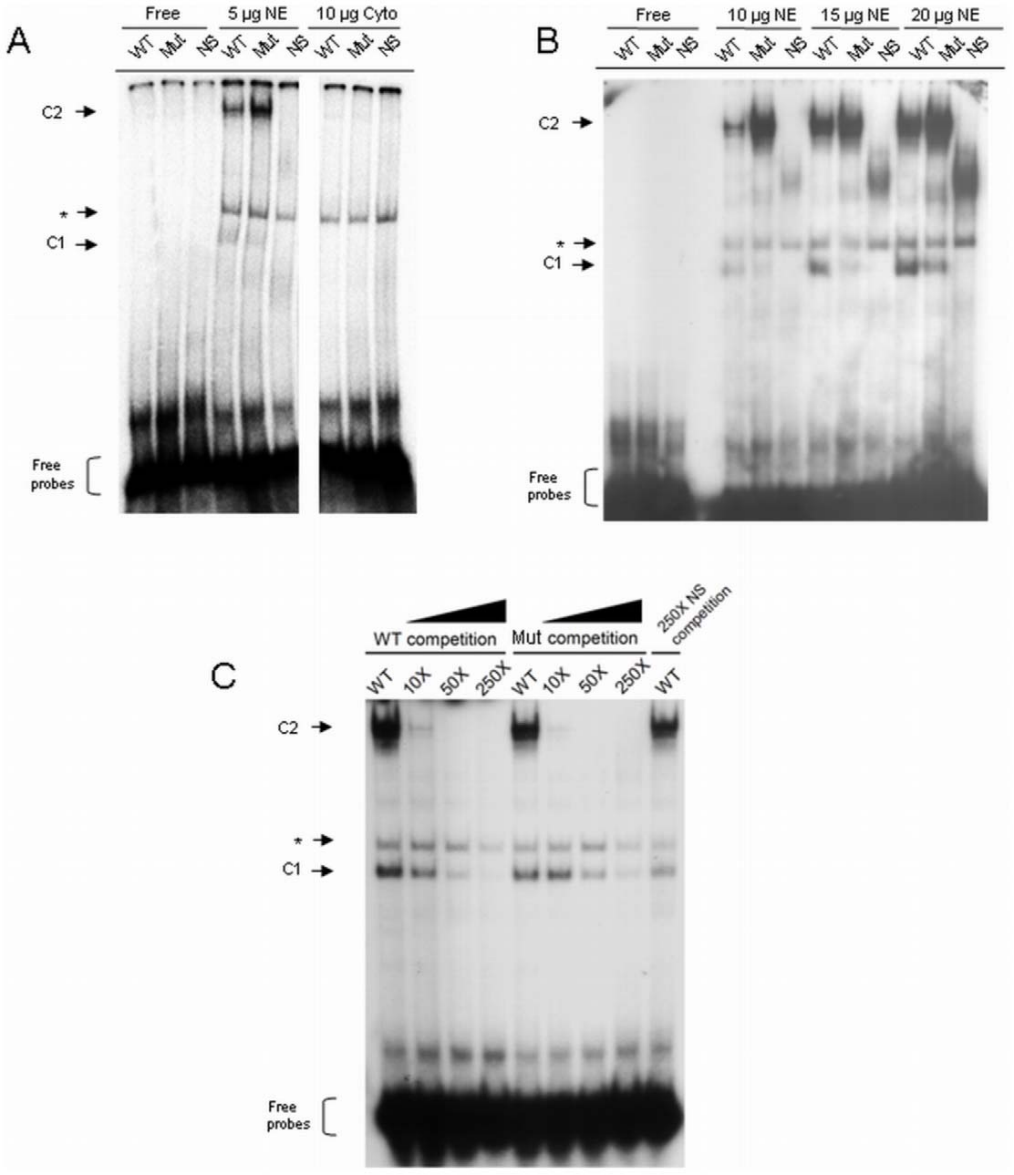
Electrophoretic mobility shift assay of wild-type and G1613A NRE oligos. (**A**) Binding of HuH7 nuclear and cytosolic fractions to the wild-type and mutant NRE oligos. The radioactive labeled oligos were titrated with 5 µg nuclear extracts and the complexes were analyzed by native polyacrylamide gel electrophoresis (PAGE). The cytosolic extracts and non-specific probes were used as negative controls. The complexes are shown as C1 and C2. The non-specific complex is indicated with an asterisk. (**B**) Titration of the WT and Mut oligos against increasing amount of nuclear extracts. Various amounts of nuclear extracts were used to bind the WT and Mut oligos and analyzed by native PAGE. (**C**) The competitive binding of protein complexes against unlabeled wild-type and mutant NRE oligos. The complexes were titrated against various amounts of unlabeled oligos (10×, 50× and 250×) and the displacement of the labeled oligos from the complexes was analyzed by native PAGE. NE: Nuclear Extract; Cyto: cytosolic fraction; WT: Wild-type oligos; Mut: Mutant oligos; NS: Non-specific oligos; C1: Protein complex I; C2: Protein complex II; *: non-specific complex.

To gain insight into the molecular effect of the G1613A mutation, NRE DNA binding with nuclear proteins was studied using EMSA. As shown in Figure 5A, two specific DNA-protein complexes (C1 and C2) were observed when the NRE oligos were incubated with nuclear but not cytosolic extracts, suggesting that the promoter interacts with the nuclear proteins in the cells. Moreover, the DNA binding to the nuclear proteins showed a dosage-dependent manner (Figure 5A and 5B). Intriguingly, the formation of C1 complexes is more favorable to wild-type than mutant whereas that of C2 complexes is more favorable to mutant. In order to further study their affinity and specificity, we titrated the complexes against increasing concentration of non-radioactive DNA. As shown in Figure 5C, DNA of C1 complex was displaced by 50× (wild-type) and 250× (mutant) non-radioactive oligos. On the other hand, DNA of C2 complex was displaced readily by 10× non-radioactive mutant DNA. Our results implied that two different binding mechanisms are involved in NRE in the context of G1613A mutation, and the mutation can modify the binding of two nuclear protein complexes to NRE region.

### RFX1 protein binds on the NRE and transactivates the core promoter activity corresponds to the G1613A mutation

**Figure 6:**
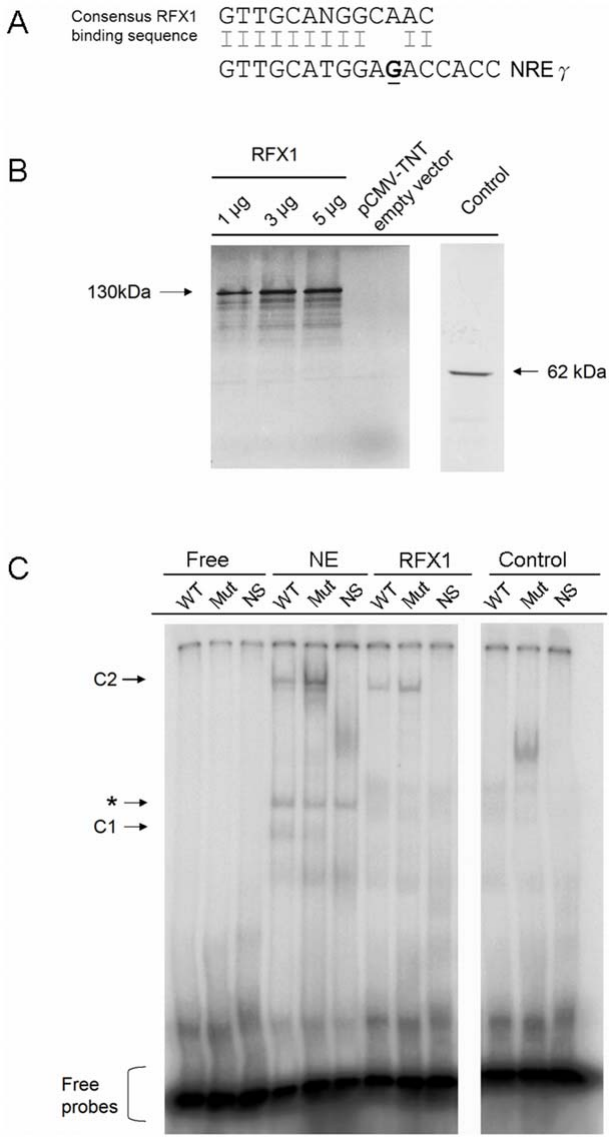
Binding of recombinant RFX1 to wild-type and G1613A mutant NRE. (**A**) Sequence alignment of the consensus binding sequence of RFX1 and the NREγ region. The consensus sequence of RFX1 was aligned with the sequence of the NREγ region (nt. 1605–1617). The underlined ‘G’ corresponds to the nt.1613 position in our study. (**B**) *In vitro* expression of RFX1 using radioactive ^35^S methionine. 1–5 µg of RFX1-expression construct was used for each reaction. The *in vitro* synthesized RFX1 protein was revealed using 10% SDS-PAGE and the signals were detected by autoradiography for 2 days. RFX1 of apparent size of 130 kDa was successfully expressed. The smear observed may be due to the degradation of proteins of large size. No band was shown in the empty vector control. The 62 kDa protein in the control correspond to the luciferase protein expressed from a control plasmid provided by the kit. (**C**) Electrophoretic mobility shift assay of Wt and Mut radioactive labeled oligos with recombinant RFX1 protein. The complexes were analyzed by native polyacrylamide gel electrophoresis. Non-specific oligos and *in vitro* transcribed luciferase protein were used as negative control. The labeling is depicted as previously described.

The NRE contains three subregions which act synergistically to suppress core promoter activity. NREγ (nt. 1605–1625) is one of these subregions that is active in HuH7 cells [14]. RFX1 is one of the transcription activator that was shown to bind NREγ on the core promoter [14], [31]. Moreover, the consensus binding sequences of RFX1 share extremely high homology with the sequence of NREγ subregion, from nt. 1605 to 1617 [26], [32], [33], [34] (Figure 6A). Intriguingly, the G1613A mutation on the NREγ site further matches the consensus RFX1 binding sequence, which is consistent to our result that the C2 complex showed higher affinity to mutant than wild-type DNA. In order to further confirm this result, we *in vitro* synthesized the RFX1 protein using rabbit reticulocyte lysate which showed a 130 kDa on SDS-PAGE (Figure 6B). Then we performed EMSA using the *in vitro* synthesized protein and NRE probes. As shown in Figure 6C, RFX1 shifted the wild-type and mutant NRE probe to the position correspond to that in the complex C2, suggesting that the C2 complex is possibly the RFX1-DNA complex. This only occurred in the NRE probes but not in the NS oligos and control, indicating the specificity between RFX1 and the DNA. Moreover, the G1613A mutation can alter the binding of RFX1 binding to NRE, in which that the RFX1 protein favors for the A1613 mutant then the wild-type. This implied that the RFX1 could possibly be one of the protein complexes which binding to NRE can be modulated by G1613A mutation.

**Figure 7:**
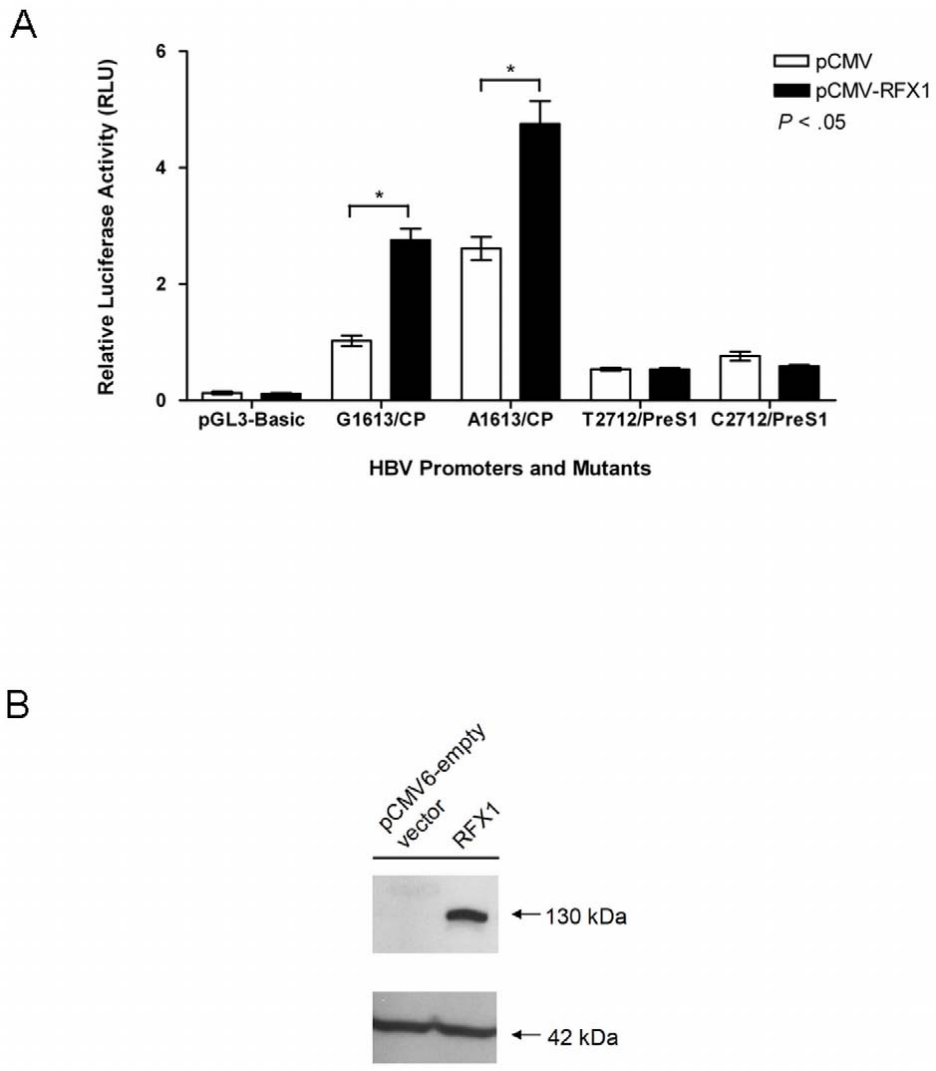
The effect of RFX1 on the HBV promoter activity. (**A**) The relative luciferase activities of the HBV promoters were measured after the overexpression of RFX1 (48 hr) in HuH7 cells. The pCMV, PreS1 promoters and pGL3-basic were used as negative controls. Results were presented as relative luciferase activity in 3 independent experiments with triplicates (mean ± S. D.) after normalizing to the co-transfected *Renilla* luciferase activities. CP: HBV Core promoter; PreS1: HBV PreS1 promoter. (**B**) Western blot of the RFX1 protein. The RFX1 protein (130 kDa) was probed with anti-myc antibody after 48 hours of transfection. β-actin (42 kDa) was the internal control.

To further investigate the role of the RFX1 on HBV core promoter via G1613A mutation, C-terminally myc-tagged RFX1 protein was overexpressed in HuH7 cells. As shown in Figure 7A, under the physiological level of RFX1, the mutated core promoter was more active when compared to wild-type. However, when RFX1 was overexpressed, it enhanced the activity of the wild-type promoter to the level of mutant, and further enhanced the mutant promoter activity. Notify that the protein had no effect on the PreS1 promoter and empty-vector control, indicating the transactivation effect was specific to core promoter. The overexpression of the RFX1 was confirmed by immunoblotting using mouse anti-myc antibody (Figure 7B). These results suggest that RFX1 plays a significant role on regulating HBV core promoter via G1613A mutation on NRE.

## Discussion

Infection of hepatitis B virus (HBV) causes acute and chronic hepatitis and is closely associated with the development of cirrhosis and hepatocellular carcinoma (HCC). To improve the survival of patients with chronic HBV infection, prognosis of the risk of cancer development will be essential because of the lack of effective drug treatments. Previously, we showed that the G1613A mutation in the HBV core promoter is a hotspot in HCC patients, which could be a potential biomarker for risk evaluation of HCC. G1613A mutation was found in HCC patients [35], particularly associates to subgenotype Cs as we demonstrated in this study. Persistent elevation of serum HBV DNA level in the 10^4^–10^7^ ranges has long considered increasing the risk of HCC [30]. Moreover, a recent report showed that high HBV viral load (more than 6 log copies/ml) is significantly associated with HCC in both univariate and multivariate analysis [17], [23], [36], [37], [38], [39], [40], [41]. In this report, we showed that the G1613A mutation is associated to a viral load of more than 6 log copies/ml in female carriers, indicating that there may be a link between this mutation and the HBV-related HCC development through the enhanced viral load. However, the reason for the discrepancy between sexes is still not clear. Moreover, we further investigated the functional consequences of the G1613A mutation in the context of 1.3× full-length replicative competent HBV genomes and provide a possible molecular mechanism of the G1613A mutation and resulted phenotype of the virus.

Our work demonstrated that the G1613A mutation leads to the suppression of HBeAg secretion. Downregulation of HBeAg results in the moderation of HBcAg-specific liver injury and viral persistency during chronic infection, leading to severe liver disease in various studies [23], [41], [42], [43], [44]. Emergence of HBeAg negative/reduced mutant during chronic infection can also lead to more active disease on liver histology, especially when the mutant becomes predominant with high viral load in serum [45]. In our results, the phenotype of the G1613A mutation which includes the suppression of HBeAg secretion and the enhancement of viral DNA production may indicate a more aggressive disease stage of liver introduced by this mutation.

Our result also demonstrated that there was no correlation between the level of HBsAg and the G1613A mutation. HBsAg has been a surrogate marker for covalently closed circular (ccc) DNA, the key transcriptional template for HBV RNA production that responsible for the viral persistence [46], [47], [48]. However, most studies investigating the association between serum HBsAg and cccDNA have focused mainly on patients with a positive HBeAg [49]. Recently, it has been reported that HBsAg cannot reflect the replication efficiency of HBV in patients with HBeAg-negative chronic hepatitis B [12], [13], [50], [51], [52]. This may be explained by the massive production of HBsAg from the cells as subviral quasispheral or filamentous lipoprotein particles without the component of core capsid and viral DNA, in addition to being incorporated into virion envelope.

A number of studies showed that mutations in NRE destroyed most of its activity and increased the core promoter activity [53]. However, the functional consequences of G1613A mutation are not yet to be determined. We demonstrated for the first time that the nt. 1613 is the hotspot of mutation within the NRE region. Moreover, we found two main protein complexes that associated with NRE sequence of core promoter and showed differential binding affinity towards the wild-type and mutant NRE sequences, suggesting the possible role of the G1613A mutation on regulating core promoter activity, and hence modulating viral replication and protein secretion. We further identified a transactivator of the HBV enhancer I, RFX1 [54], binds to the G1613A mutant with higher affinity than the wild-type sequence and possesses the trans-activating effect to enhance the core promoter activity in liver cells. Intriguingly, the G1613A mutated NRE sequence share higher homology with the consensus RFX1 binding sequence than the wild-type sequence, indicating the higher affinity of the mutant NRE to the protein leads to the transactivation of the core promoter activity.

As the mutation alters the core promoter activity, however, the levels of HBV transcripts were not significantly affected. All HBV mRNAs have a common polyadenylation site and the highly conserved regions of PRE at the 3′ end. Intriguingly, the minimal region of the PRE was shown within nt. 1151–1684 [20] in which our G1613A mutation is found. The PRE has been reported to be involved in the regulation of HBV mRNAs including RNA splicing [19], RNA stability [15], [16], [54] and nuclear export [18]. Computational analysis of the PRE sequence revealed three putative functional conserved elements whereas one of them (nt. 1510–1620) contains a conserved and defined RNA secondary structure [55]. The G1613A mutation within this region may possibly disrupts this secondary structure and hence, affect the function of PRE. Indeed, glyceraldehyde-3-phosphate dehydrogenase (GAPDH) [37] and polypyrimidine tract binding protein (PTB) [38] have been shown to bind to the full length PRE (nt. 1239–1805) except the region nt. 1375–1487 [20]. The functional role of these proteins binding to PRE was believed to increase HBV gene expression at the posttranscriptional level, probably by increasing viral RNA export out of the nucleus. Moreover, PTB is known to contribute to RNA stability [39] and translation initiation [37], [56]. It also represses pgRNA splicing and facilitates the export of unspliced subgenomic RNAs. Whether the G1613A mutation affect the binding to these proteins deserves to be investigated in the future. Interestingly, although all HBV RNAs contain the same PRE sequence, it seems that the function of the PRE is differentially regulated depending on the context of the RNA sequences [15], [54], [57]. For example, the PRE is considered to enhance HBV surface protein expression by facilitating nuclear export of subgenomic RNAs [20]. In contrast, the PRE has been shown to contributed to pgRNA splicing and stability [58]. However, its role in the metabolism of the pgRNA is not well understood. Nevertheless, in our study, the G1613A mutation affects not only at promoter level but also possibly posttranscriptionally, suggesting the tightly regulated processes of HBV replication.

In summary, we demonstrated that the G1613A mutation, a hotspot mutation on NRE found in HCC patients suppresses the HBeAg secretion and enhances the viral load, which may possibly lead to a more active hepatitis and the risk to HCC. Moreover, we demonstrated that RFX1 plays a significant role on transactivating HBV core promoter activity with the G1613A mutation and suggested a possible molecular linkage between this mutation and the resulted phenotype of the virus.

## References

[1] Beasley RP, Hwang LY, Lin CC, Chien CS (1981). Hepatocellular carcinoma and hepatitis B virus. A prospective study of 22 707 men in Taiwan.. Lancet.

[2] Kao JH (2002). Hepatitis B viral genotypes: clinical relevance and molecular characteristics.. J Gastroenterol Hepatol.

[3] Lai CL, Ratziu V, Yuen MF, Poynard T (2003). Viral hepatitis B.. Lancet.

[4] Chan HL, Tsui SK, Tse CH, Ng EY, Au TC (2005). Epidemiological and virological characteristics of 2 subgroups of hepatitis B virus genotype C.. J Infect Dis.

[5] Guidotti LG, Matzke B, Schaller H, Chisari FV (1995). High-level hepatitis B virus replication in transgenic mice.. J Virol.

[6] Kramvis A, Kew MC (1999). The core promoter of hepatitis B virus.. J Viral Hepat.

[7] Orito E, Ichida T, Sakugawa H, Sata M, Horiike N (2001). Geographic distribution of hepatitis B virus (HBV) genotype in patients with chronic HBV infection in Japan.. Hepatology.

[8] Moolla N, Kew M, Arbuthnot P (2002). Regulatory elements of hepatitis B virus transcription.. J Viral Hepat.

[9] Yu X, Mertz JE (1997). Differential regulation of the pre-C and pregenomic promoters of human hepatitis B virus by members of the nuclear receptor superfamily.. J Virol.

[10] Ganem D, Prince AM (2004). Hepatitis B virus infection–natural history and clinical consequences.. N Engl J Med.

[11] Yuh CH, Chang YL, Ting LP (1992). Transcriptional regulation of precore and pregenomic RNAs of hepatitis B virus.. J Virol.

[12] Chen M, Ou JH (1995). Cell type-dependent regulation of the activity of the negative regulatory element of the hepatitis B virus core promoter.. Virology.

[13] Lo WY, Ting LP (1994). Repression of enhancer II activity by a negative regulatory element in the hepatitis B virus genome.. J Virol.

[14] Buckwold VE, Chen M, Ou JH (1997). Interaction of transcription factors RFX1 and MIBP1 with the gamma motif of the negative regulatory element of the hepatitis B virus core promoter.. Virology.

[15] Huang J, Liang TJ (1993). A novel hepatitis B virus (HBV) genetic element with Rev response element-like properties that is essential for expression of HBV gene products.. Mol Cell Biol.

[16] Huang ZM, Yen TS (1994). Hepatitis B virus RNA element that facilitates accumulation of surface gene transcripts in the cytoplasm.. J Virol.

[17] Huang ZM, Yen TS (1995). Role of the hepatitis B virus posttranscriptional regulatory element in export of intronless transcripts.. Mol Cell Biol.

[18] Panjaworayan N, Payungporn S, Poovorawan Y, Brown CM (2010). Identification of an effective siRNA target site and functional regulatory elements, within the hepatitis B virus posttranscriptional regulatory element.. Virol J.

[19] Ehlers I, Horke S, Reumann K, Rang A, Grosse F (2004). Functional characterization of the interaction between human La and hepatitis B virus RNA.. J Biol Chem.

[20] Heise T, Sommer G, Reumann K, Meyer I, Will H (2006). The hepatitis B virus PRE contains a splicing regulatory element.. Nucleic Acids Res.

[21] Gunther S, Sommer G, Iwanska A, Will H (1997). Heterogeneity and common features of defective hepatitis B virus genomes derived from spliced pregenomic RNA.. Virology.

[22] Rosmorduc O, Petit MA, Pol S, Capel F, Bortolotti F (1995). In vivo and in vitro expression of defective hepatitis B virus particles generated by spliced hepatitis B virus RNA.. Hepatology.

[23] Sommer G, van Bommel F, Will H (2000). Genotype-specific synthesis and secretion of spliced hepatitis B virus genomes in hepatoma cells.. Virology.

[24] Su TS, Lai CJ, Huang JL, Lin LH, Yauk YK (1989). Hepatitis B virus transcript produced by RNA splicing.. J Virol.

[25] Terre S, Petit MA, Brechot C (1991). Defective hepatitis B virus particles are generated by packaging and reverse transcription of spliced viral RNAs in vivo.. J Virol.

[26] Sung JJ, Tsui SK, Tse CH, Ng EY, Leung KS (2008). Genotype-specific genomic markers associated with primary hepatomas, based on complete genomic sequencing of hepatitis B virus.. J Virol.

[27] Chan HL, Tse CH, Mo F, Koh J, Wong VW (2008). High viral load and hepatitis B virus subgenotype ce are associated with increased risk of hepatocellular carcinoma.. J Clin Oncol.

[28] Mendy ME, Kaye S, van der Sande M, Rayco-Solon P, Waight PA (2006). Application of real-time PCR to quantify hepatitis B virus DNA in chronic carriers in The Gambia.. Virol J.

[29] Wang YX, Xu X, Luo C, Ma ZM, Jiang HL (2007). Mutational analysis revealed that conservation of hepatitis B virus reverse transcriptase residue 306 (rtP306) is crucial for encapsidation of pregenomic RNA.. FEBS Lett.

[30] Wong VW, Chan SL, Mo F, Chan TC, Loong HH (2010). Clinical scoring system to predict hepatocellular carcinoma in chronic hepatitis B carriers.. J Clin Oncol.

[31] Reinhold W, Emens L, Itkes A, Blake M, Ichinose I (1995). The myc intron-binding polypeptide associates with RFX1 in vivo and binds to the major histocompatibility complex class II promoter region, to the hepatitis B virus enhancer, and to regulatory regions of several distinct viral genes.. Mol Cell Biol.

[32] Cheng Y, Seet BL, Ong CS, Wasser S, Tan TM (2006). Are in vitro hepatitis B core promoter mutations important for clinical alterations in viral load?. Antiviral Res.

[33] Shinkai N, Tanaka Y, Ito K, Mukaide M, Hasegawa I (2007). Influence of hepatitis B virus X and core promoter mutations on hepatocellular carcinoma among patients infected with subgenotype C2.. J Clin Microbiol.

[34] Takahashi K, Akahane Y, Hino K, Ohta Y, Mishiro S (1998). Hepatitis B virus genomic sequence in the circulation of hepatocellular carcinoma patients: comparative analysis of 40 full-length isolates.. Arch Virol.

[35] Liu TT, Fang Y, Xiong H, Chen TY, Ni ZP (2008). A case-control study of the relationship between hepatitis B virus DNA level and risk of hepatocellular carcinoma in Qidong, China.. World J Gastroenterol.

[36] Terazawa S, Kojima M, Yamanaka T, Yotsumoto S, Okamoto H (1991). Hepatitis B virus mutants with precore-region defects in two babies with fulminant hepatitis and their mothers positive for antibody to hepatitis B e antigen.. Pediatr Res.

[37] Zang WQ, Li B, Huang PY, Lai MM, Yen TS (2001). Role of polypyrimidine tract binding protein in the function of the hepatitis B virus posttranscriptional regulatory element.. J Virol.

[38] Huang ZM, Zang WQ, Yen TS (1996). Cellular proteins that bind to the hepatitis B virus posttranscriptional regulatory element.. Virology.

[39] Jang SK, Wimmer E (1990). Cap-independent translation of encephalomyocarditis virus RNA: structural elements of the internal ribosomal entry site and involvement of a cellular 57-kD RNA-binding protein.. Genes Dev.

[40] Liang TJ, Hasegawa K, Rimon N, Wands JR, Ben-Porath E (1991). A hepatitis B virus mutant associated with an epidemic of fulminant hepatitis.. N Engl J Med.

[41] Milich D, Liang TJ (2003). Exploring the biological basis of hepatitis B e antigen in hepatitis B virus infection.. Hepatology.

[42] Chan HL, Tsang SW, Liew CT, Tse CH, Wong ML (2002). Viral genotype and hepatitis B virus DNA levels are correlated with histological liver damage in HBeAg-negative chronic hepatitis B virus infection.. Am J Gastroenterol.

[43] Kumar M, Sarin SK, Hissar S, Pande C, Sakhuja P (2008). Virologic and histologic features of chronic hepatitis B virus-infected asymptomatic patients with persistently normal ALT.. Gastroenterology.

[44] Zacharakis G, Koskinas J, Kotsiou S, Tzara F, Vafeiadis N (2008). The role of serial measurement of serum HBV DNA levels in patients with chronic HBeAg(-) hepatitis B infection: association with liver disease progression. A prospective cohort study.. J Hepatol.

[45] Tuttleman JS, Pourcel C, Summers J (1986). Formation of the pool of covalently closed circular viral DNA in hepadnavirus-infected cells.. Cell.

[46] Werle-Lapostolle B, Bowden S, Locarnini S, Wursthorn K, Petersen J (2004). Persistence of cccDNA during the natural history of chronic hepatitis B and decline during adefovir dipivoxil therapy.. Gastroenterology.

[47] Wursthorn K, Lutgehetmann M, Dandri M, Volz T, Buggisch P (2006). Peginterferon alpha-2b plus adefovir induce strong cccDNA decline and HBsAg reduction in patients with chronic hepatitis B.. Hepatology.

[48] Chan HL, Wong VW, Tse AM, Tse CH, Chim AM (2007). Serum hepatitis B surface antigen quantitation can reflect hepatitis B virus in the liver and predict treatment response.. Clin Gastroenterol Hepatol.

[49] Lin LY, Wong VW, Zhou HJ, Chan HY, Gui HL (2010). Relationship between serum hepatitis B virus DNA and surface antigen with covalently closed circular DNA in HBeAg-negative patients.. J Med Virol.

[50] Gerlach KK, Schloemer RH (1992). Hepatitis B virus C gene promoter is under negative regulation.. Virology.

[51] Sun CT, Lo WY, Wang IH, Lo YH, Shiou SR (2001). Transcription repression of human hepatitis B virus genes by negative regulatory element-binding protein/SON.. J Biol Chem.

[52] Park GT, Yi YW, Choi CY, Rho HM (1997). A negative regulatory element and its binding protein in the upstream of enhancer II of hepatitis B virus.. DNA Cell Biol.

[53] Siegrist CA, Durand B, Emery P, David E, Hearing P (1993). RFX1 is identical to enhancer factor C and functions as a transactivator of the hepatitis B virus enhancer.. Mol Cell Biol.

[54] Donello JE, Beeche AA, Smith GJ, Lucero GR, Hope TJ (1996). The hepatitis B virus posttranscriptional regulatory element is composed of two subelements.. J Virol.

[55] Zang WQ, Fieno AM, Grant RA, Yen TS (1998). Identification of glyceraldehyde-3-phosphate dehydrogenase as a cellular protein that binds to the hepatitis B virus posttranscriptional regulatory element.. Virology.

[56] Li B, Yen TS (2002). Characterization of the nuclear export signal of polypyrimidine tract-binding protein.. J Biol Chem.

[57] Huang ZM, Yen TS (1993). Dysregulated surface gene expression from disrupted hepatitis B virus genomes.. J Virol.

[58] Wollerton MC, Gooding C, Wagner EJ, Garcia-Blanco MA, Smith CW (2004). Autoregulation of polypyrimidine tract binding protein by alternative splicing leading to nonsense-mediated decay.. Mol Cell.

